# Effect of induced dNTP pool imbalance on HIV-1 reverse transcription in macrophages

**DOI:** 10.1186/s12977-019-0491-0

**Published:** 2019-10-26

**Authors:** Caitlin Shepard, Joella Xu, Jessica Holler, Dong-Hyun Kim, Louis M. Mansky, Raymond F. Schinazi, Baek Kim

**Affiliations:** 1 Department of Pediatrics, School of Medicine, Emory University, 1760 Haygood Drive E432, Atlanta, GA 30322 USA; 2 School of Pharmacy, Kyung-Hee University, Seoul, South Korea; 3 Institute for Molecular Virology, University of Minnesota, Minneapolis, MN USA; 4 Center for Drug Discovery, Children’s Healthcare of Atlanta, Atlanta, GA USA

**Keywords:** HIV-1, Reverse transcription, SAMHD1, dNTP pool imbalance, Mutagenesis, Macrophages

## Abstract

**Background:**

Terminally differentiated/nondividing macrophages, a key target cell type of HIV-1, harbor extremely low dNTP concentrations established by a host dNTP triphosphohydrolase, SAM domain and HD domain containing protein 1 (SAMHD1). We tested whether the induction of dNTP pool imbalance can affect HIV-1 replication in macrophages. For this test, we induced a large dNTP pool imbalance by treating human primary monocyte derived macrophages with either one or three of the four deoxynucleosides (dNs), which are phosphorylated to dNTPs in cells, to establish two different dNTP imbalance conditions in macrophages.

**Results:**

The transduction efficiency and 2-LTR circle copy number of HIV-1 GFP vector were greatly diminished in human primary macrophages treated with the biased dN treatments, compared to the untreated macrophages. We also observed the induced dNTP bias blocked the production of infectious dual tropic HIV-1 89.6 in macrophages. Moreover, biochemical DNA synthesis by HIV-1 reverse transcriptase was significantly inhibited by the induced dNTP pool imbalance. Third, the induced dNTP bias increased the viral mutant rate by approximately 20–30% per a single cycle infection. Finally, unlike HIV-1, the single dN treatment did not significantly affect the transduction of SIV_mac_239-based GFP vector encoding Vpx in macrophages. This is likely due to Vpx, which can elevate all four dNTP levels even with the single dN treatment.

**Conclusion:**

Collectively, these data suggest that the elevated dNTP pool imbalance can induce kinetic block and mutation synthesis of HIV-1 in macrophages.

## Background

It has been shown that dNTP pool imbalance in cells is one mechanistic element for mutation synthesis during genomic DNA replication [1, 2]. Differential incorporation kinetics of DNA polymerases among the four types of dNTPs during DNA synthesis are intrinsically determined by template-sequences [3], DNA polymerase types [4] as well as biased dNTP pools [5, 6]. Also, DNA polymerase pausing, induced by both template sequences and limited dNTP pools during processive DNA synthesis is known to contribute to mutation synthesis. This is because DNA polymerases tend to be more error-prone during restarts after pausing, and the pause sites often become mutational hot spots [2]. Biased dNTP pools can not only perturb processive DNA synthesis kinetics, but also facilitate frequent mutation synthesis.

Importantly, as observed in published dNTP measurements, the concentrations of the four dNTPs in cells are never equal [7, 8]. Concentrations of cellular dNTPs vary significantly, mainly depending on their cell cycle status. The cellular dNTP biosynthesis machinery is highly active in dividing cells, specifically in G1/S and S phases [9, 10]. In these phases, dNTPs are consumed for genomic DNA synthesis catalyzed by cellular replicative DNA polymerases. Cells with uncontrolled cell cycle such as cancer cells and transformed cells have elevated dNTP levels compared to normal cells [11–13]. Such elevated dNTP levels were proposed as a biomarker for cancer cells [7, 12]. The dNTP levels in nondividing cells had been postulated to be lower than dividing cells due to lack of the active dNTP biosynthesis. Indeed, the actual dNTP concentrations in human primary nondividing cells could not be measured due to the limited sensitivity of the assays to measure dNTP amounts. However, after our discovery of the uniquely efficient dNTP incorporation efficiency of HIV-1 reverse transcriptase (RT) even at low dNTP concentrations, we established the HIV-1 RT based enzymatic dNTP assay [8]. This enabled us to measure the dNTP concentration of human primary nondividing cells. Indeed, human primary monocyte-derived macrophages harbor approximately 50–100 times lower dNTP concentrations (20–50 nM) than activated/dividing CD4^+^ T cells (1–5 μM) [8].

dNTP biosynthesis enzymes such as ribonucleotide reductase (RNR) and thymidine kinases have been considered a major regulator of the cellular dNTP levels, especially in dividing cells. The recent discovery of cellular sterile alpha motif domain and histidine-aspartate domain containing protein 1 (SAMHD1), revealed its ability to hydrolyze dNTPs to deoxynucleosides (dNs) and triphosphates [14]. We previously reported that SAMHD1 depletes cellular dNTPs in nondividing macrophages and is responsible for their extremely low dNTP concentration [15]. This SAMHD1-mediated limited dNTP availability in macrophages kinetically suppresses HIV-1 reverse transcription. However, unlike HIV-1, HIV-2 and some SIV strains encode a unique viral accessory protein, viral protein X (Vpx). Vpx proteosomally degrades SAMHD1 in the nucleus and elevates cellular dNTP concentrations [16–18]. The degradation of SAMHD1 leads to the acceleration of viral replication in macrophages [19].

Lentiviruses, including HIV-1, infect activated/dividing CD4^+^T cells as well as nondividing cells, such as macrophages, during their pathogenesis [20, 21]. Even though the *K*_m_ value of HIV-1 RT is uniquely low compared with other DNA polymerases, it is above the dNTP concentrations found in macrophages [15]. This explains why SAMHD1 dNTPase suppresses HIV-1 reverse transcription kinetics of HIV-1 in macrophages. Since HIV-1 reverse transcription occurs below the *K*_m_ values of HIV-1 RT in the low dNTP pool environment of macrophages, the dNTP incorporation kinetics are directly affected by the availability/concentration of each dNTP. Therefore, it is more likely that dNTP pool imbalance at these low dNTP concentrations can induce more frequent pausing of HIV-1 RT in macrophage, compared to activated CD4^+^ T cells harboring abundant dNTP pools (above than *K*_m_ value and close to *V*_max_, [15, 22]). The dNTP pool imbalance promotes the incorporation of incorrect dNTPs and generates a mismatched primer. Since this mismatched primer is much more kinetically difficult to extend, compared to a matched primer [23, 24], the dNTP pool imbalances were hypothesized to perturb the processive HIV-1 proviral DNA synthesis and viral replication kinetics.

In this study, we established large dNTP pool imbalance in human primary monocyte-derived macrophages. We tested whether and how this induced dNTP pool imbalance in macrophages affects HIV-1 replication and mutagenesis. Our data support that the large dNTP imbalance in macrophages induces kinetic blocks and mutation synthesis of HIV-1.

## Materials and procedures

### Cells, vectors and viruses

Primary human monocytes were isolated from the peripheral blood buffy coats of 4 donors (New York Blood Center, New York, New York) by positive selection using MACS CD14^+^ (Miltenyi Biotec) beads as previously described [8]. Monocytes were pooled in an equal number per donor and were maturated into monocyte-derived macrophages (MDMs) in the presence of 15 ng/ml hGM-CSF (Miltenyi Biotec) treated at days 0 and 3 of maturation. MDMs were used at day 8 of maturation for experiments. HIV-1 89.6 viruses were prepared as previously described [25]. After 8 passages in CEMx174 cells, PCR of the beta-lactamase gene, found in the plasmid, was done on nucleic acid extracted from the supernatant. None was detected, demonstrating a plasmid-free culture. HIV-1 p24 ELISA (Advanced Bioscience Laboratories Inc.) was used for monitoring and quantitating the produced viruses. pD3HIV-GFP vector was prepared as previously described [26]. pD3HIV-GFP vector encodes the HIV-1 NL4-3 genome with the eGFP gene replacing the HIV-1 nef gene, additionally the envelope gene is deleted [8]. We transfected 293FT cells with pD3HIV-GFP and pVSV-g using polyethylenimine, and media was collected on day three for ultracentrifugation. This same method was used for the generation of pSIV_mac_239-GFP, a gift from Dr. Diaz-Griffero (Albert Einstein College of Medicine, NYC), and pNL4-3 MIG. Virus-like particles (VLPs) were generated as previously described [26].

### HIV-1 and SIV vector transduction

pD3HIV-GFP or pSIV_mac_239-GFP was transduced into macrophage cells after at least 8 days of maturation. The cells were treated for 4 h with the concentration and dN as specified in the figure legend. Next, the vector was added to the dN media and allowed to transduce for 6 days before measuring the transduction efficiency. The cells were harvested and fixed with 3.7% formaldehyde. GFP levels were measured via FACS (Miltenyi Biotec, VYB).

### Cellular dNTP measurement

Measurement was done as previously described [8]. MDMs were lysed with 60% cold methanol. Samples were vortexed, heated at 95 °C for 3 min and cellular debris were cleared by 14 K rpm centrifugation. Supernatant was dried using a SpeedVac. Pellets were resuspended in water diluted to be within linear range of the assay, 2–50%. 5′ ^32^P-end-labeled 18-mer DNA primer (5′-GTCCCTCTTCGGGCGCCA-3′, Integrated DNA Technologies) was individually annealed to one of four unique 19-mer DNA templates (3′-CAGGGAGAAGCCCGCGGTN-5′, Integrated DNA Technologies). Reactions contained 200 fmol template/primer, 4 μL of purified RT (HIV-1 HXB2), 25 mM Tris–HCl, pH 8.0, 2 mM dithiothreitol, 100 mM KCl, 5 mM MgCl2, and 10 μM oligo (dT), and cellular dNTP extracts with a final reaction volume of 20 μL. After 5 min incubation at 37 °C, each reaction was stopped with 10 μL of 40 mM EDTA and 99% (vol/vol) formamide at 95 °C for 2 min. The reaction causes the template/primer to be extended by HIV-1 reverse transcriptase, generating one additional nucleotide extension product for one of four dNTPs contained in the sample. These products are resolved on a 14% urea-PAGE gel (AmericanBio, Inc.) and analyzed using PharosFX molecular imager and Image lab software (Biorad). In this assay, the molar amount of product is equal to that of each dNTP contained in the extracted samples, which allows us to calculate and compare the dNTP concentrations for different dN treatments [8].

### HIV-1 RT mediated primer extension assay

**Fig. 1:**
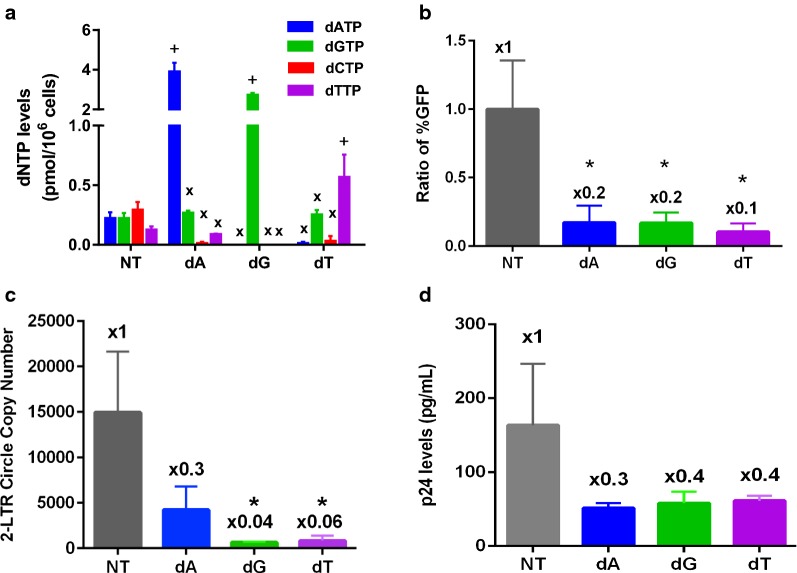
Effect of single dNTP elevation imbalances on HIV-1 infection in macrophages. All experiments were conducted in human primary monocyte-derived macrophages prepared from four healthy donors. dA and dT treatments were at 2.5 mM and dG treatment was at 1 mM, due to solubility limits. **a** dNTP levels were measured after macrophages were treated with a single dN treatment. After 12 h single dN treatment, the dNTP levels in these cells were determined by the RT-based dNTP assay [8]. “+” indicates the dNTPs of the corresponding treated dNs, and “x” indicates untreated dNTPs. *NT* No dN treatment. **b** HIV-1 vector transduction efficiency was measured after macrophages were pretreated with dNs for 4 h, and then transduced with an equal amount of HIV-GFP vector. The transduced cells were collected after 6 days, and the percent of the GFP^+^ cells was determined by FACS. The ratio of the vector transduction efficiency at each single dN treatment was normalized to the transduction efficiency with no dN treatment, which gave ~ 10% transduction efficiency. See Additional file 1: Table S2 for Raw %GFP numbers. **c** Reverse transcription kinetics was determined by measuring the copy numbers of HIV-1 2-LTR circle DNAs in the cellular genomic DNAs isolated from the treated macrophages. **d** p24 antigen levels were measured by ELISA. Macrophages were treated with single dNs for 4 h followed by dual tropic HIV-1 89.6 infection. Supernatant was collected 6 days after infection. Supernatant was used for a HIV-1 p24 ELISA. The measurement ratios were normalized with the no treatment (NT) conditions, and marked at the top of each readout. The data in this figure are the mean of three independent experiments and error bars represent the standard deviation from the mean. *p-value < 0.05

The primer extension assay was modified from a previously described assay [27]. A template/primer was prepared by annealing a 5′ ^32^P-end-labeled 18-mer DNA primer (5′-CGCGCCGAATTCCCGCTA-3′, Integrated DNA Technologies) to a fourfold excess of 26-mer template DNA (5′-CTAGCTAGTAGCGGGAATTCGGCGCG-3′, Integrated DNA Technologies). Each reaction contained 2 nM T/P, RT (311.3 nM), and dNTPs at the concentrations summarized in Fig. 1 and specified in Additional file 1: Table S1. After 5 min incubation at 37 °C each reaction was stopped with 10 μL of 40 mM EDTA and 99% (vol/vol) formamide at 95 °C for 10 min. As shown previously [27], these conditions allow for multiple rounds of primer extension. These products are resolved on a 14% urea-PAGE gel (AmericanBio, Inc.) and analyzed using Typhoon Imager (GE Healthcare).

### Quantitative 2-LTR circle DNA PCR

Macrophages pooled from 4 healthy donors with dN pretreated for 4 h were transduced with pD3HIV-GFP in triplicate. The cells were harvested at 6 days post transduction. The total cellular DNA was extracted using a genomic extraction kit (Promega). Primers for the amplification of the HIV-1 2-LTR circle DNA were described previously [28]. The forward primer anneals 75 bp upstream from the 3′ end of the 5-LTR region of NL4-3 (5-GTGCCCGTCTGTTGTGTGACT-3), the reverse primer anneals 33 bp downstream of the 5′ end of the 3-LTR region (5-CTTGTCTTCTTTGGGAGTGAATTAGC-3), and the probe (5-6-carboxyfluorescein-TCCACACTGACTAAAAGGGTCTGAGGGATCTCT-carboxytetramethylrhodamine-3) anneals at the junction of 5′–3′-LTR region. The amplification was performed with the forward primer (0.5 μM), the reverse primer (0.5 μM), the probe (0.25 μM), and approximately 200 ng of the extracted cellular DNA using LightCycler 480 Probes master kit (Roche). PCRs were initially incubated at 95 °C for 5 min. Each of the 45 cycles for PCR was then performed at 95 °C for 10 s, 60 °C for 30 s and 72 °C for 1 s. Finally, reactions were cooled at 40 °C for 30 s. Standard curves for the quantification of 2-LTR were generated by serial dilution of a known concentration of 2-LTR DNA. Standard curve was linear within 10^1^ to 10^6^ copies of 2-LTR DNA.

### HIV-1 89.6 infection and infectivity of produced viruses

For infection, macrophages were pretreated with dN treatment for 4 h followed by dual tropic HIV-1 89.6 infection. The virus was left on overnight and washed off the next morning. New dN media was supplied. The cells were always under the dN media condition. Supernatant was collected 6 days after infection. Supernatant was used for a HIV-1 p24 ELISA (Advanced Bioscience Laboratories Inc.) under the imbalanced dN conditions.

### HIV-1 mutation rate measurement

For single-cycle infections, HIV-1 envelope-deficient vector pNL4-3 MIG (gift from Louis Mansky lab, University of Minnesota, Minneapolis, Minnesota, [29, 30]) was used. pNL4-3 MIG expresses mCherry and enhanced green fluorescent protein (EGFP). After transducing dN treated macrophage with pMIG, mutant frequency analysis was done by FACS (Miltenyi Biotec, VYB). Mutant frequency was determined based on the percentage of single positive cells for each population compared to the total number of infected cells. The equation used was (mCherry^+^EGFP^−^cells + mCherry^−^EGFP^+^ cells) divided by total infected cells.

## Results

### Establishment of a single dNTP elevation imbalance in macrophages

Human primary monocyte-derived macrophages harbor extremely low dNTP concentrations (20–40 nM) [8]. These dNTP concentrations are induced by the dNTP triphosphohydrolase (dNTPase) activity of the host SAMHD1 protein [14–16]. These dNTP concentrations, lower than the *K*_m_ values of HIV-1 RT (100–200 nM) [15], can restrict HIV-1 reverse transcription kinetics in macrophages. First, we established biased dNTP pools under low dNTP concentrations by treating human primary monocyte-derived macrophages with one of the four dNs, which are converted to dNTPs in cells. The dNTP levels of the treated and untreated macrophages were determined by the RT-based dNTP assay [8]. It is known from our previous work that when macrophages were treated with all four dNs, all four dNTP levels are elevated above the *K*_m_ value of HIV-1 RT, which promotes HIV-1 reverse transcription and infection in macrophages [15]. This was also confirmed as shown in Additional file 1: Figure S1A. In this study, when macrophages were treated with only single dNs (Fig. 1a), we established a “single dNTP elevation imbalance” by two different effects: (1) the single corresponding dNTP levels were highly elevated (see “+”) and (2) the other three dNTPs actually were further lowered or approximately equal to the levels compared to the untreated (NT) macrophages (see “x”). This confirmed the generation of large dNTP pool imbalances.

### Effect of single dNTP elevation imbalances on HIV-1 replication in macrophages

Next, we investigated the effect of the single dNTP elevation imbalances on HIV-1 replication in human primary macrophages. First, we pretreated macrophages with these biased, single dNs, and transduced these cells with HIV-1 GFP vector. The vector transduction efficiency was determined by FACS readout of GFP expressing cells at day 6 post transduction. First, as previously reported [15], the treatment with all four dNs, which elevates all four dNTPs and overcomes SAMHD1-mediated dNTP depletion, greatly enhanced HIV-1 vector transduction efficiency and production of infectious dual tropic HIV-1 89.6 (Additional file 1: Figure S1). However, the biased dN treatments, which established larger dNTP pool imbalances, significantly reduced the HIV-1 vector transduction efficiency in comparison to no treatment (NT; Fig. 1b). Next, we measured the HIV-1 2-LTR circle DNA copy numbers in the vector transduced cells to test whether the HIV-1 vector transduction reduction is due to the effect of the dNTP pool imbalances on completed viral reverse transcription. As shown in Fig. 1c, the biased dN treatment also decreased the 2-LTR circle copy numbers, compared to the untreated macrophages. These data suggest that the biased dNTP pools reduced HIV-1 vector transduction, likely by inhibiting the completion of the reverse transcription step of HIV-1 in macrophages.

Next, we tested the same effect of the dNTP pool imbalances on HIV-1 infection by using infectious dual tropic HIV-1 89.6. The dN pretreated macrophages were infected with HIV-1 89.6, and the p24 levels in the media collected at day 6 post infection were measured. The three different elevation bias dN treatments reduced the p24 levels in the media (Fig. 1d). Overall, the data shown in Fig. 1 demonstrates that the induced single dNTP elevation imbalances inhibit HIV-1 replication in macrophages.

### Establishment and effect of a single dNTP depletion imbalance in macrophages

**Fig. 2:**
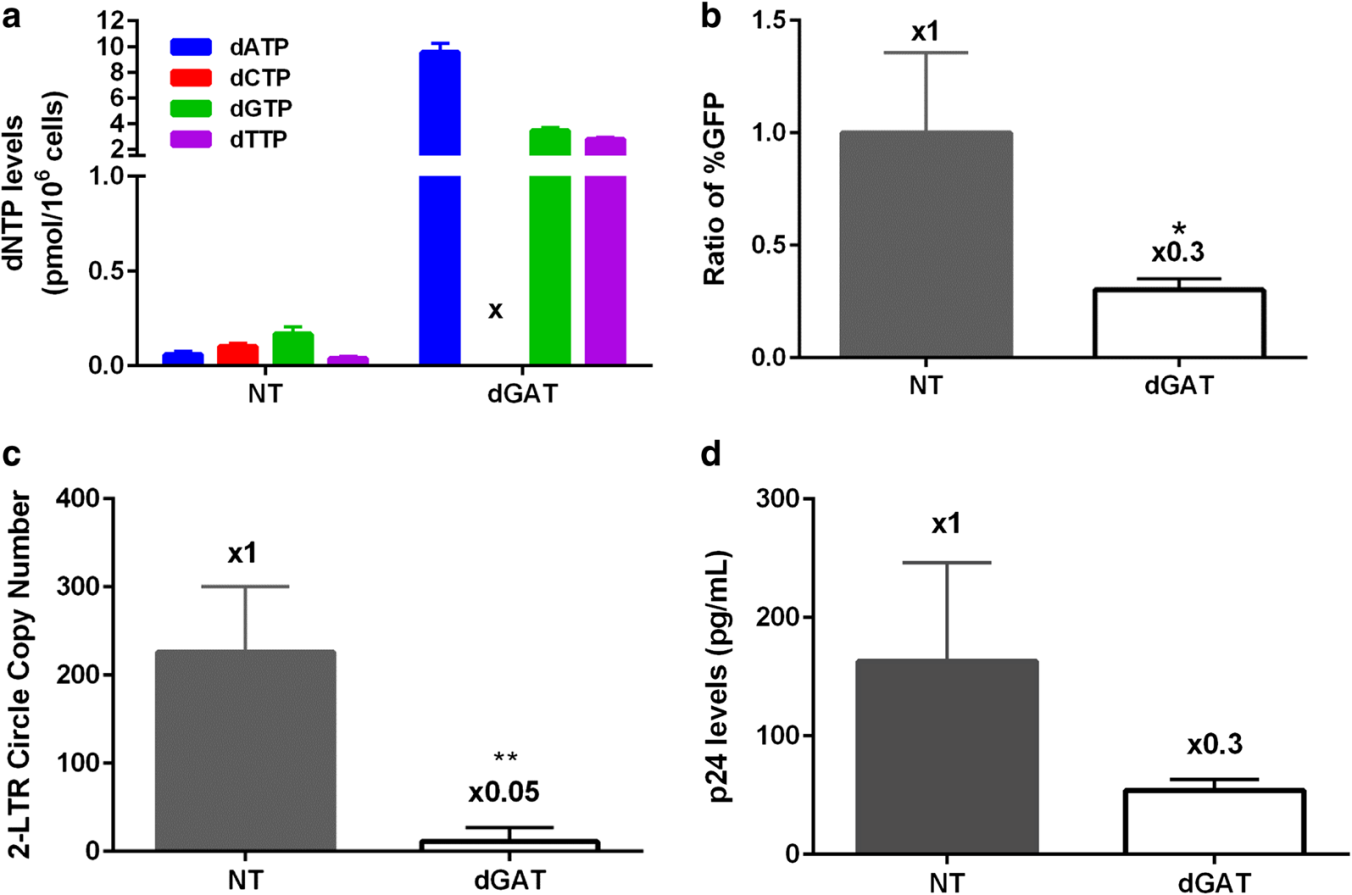
Effect of single dNTP depletion imbalance on HIV-1 infection in macrophages. Human primary monocyte-derived macrophages prepared from four healthy donors were cultured with and without a mixture of dA (2.5 mM), dT (2.5 mM) and dG (1 mM), and used for each experiment: **a** dNTP level measurement, **b** transduction efficiency measurement of HIV-1 GFP vector, **c** 2-LTR circle DNA copy number assay from the GFP vector transduced cells, and **d** p24 level assay with infectious HIV-1 89.6 were conducted as described in Fig. 1. “x” indicates untreated dNTPs. *NT* no dN treatment. The measurement ratios were normalized with the no treatment (NT) conditions, and marked at the top of each readout. The data are the mean of three independent experiments and error bars represent the standard deviation from the mean. *p-value < 0.05, **p-value < 0.01

Next, we tested whether the treatment of macrophages with only three kinds of dNs can also establish dNTP pool imbalances where only one of the four dNTPs becomes limited. For this test, we treated macrophages with three of four dNs, a mixture of dA, dG and dT (excluding dC). As shown in Fig. 2a, when macrophages were treated with a mixture of dA, dG and dT, we observed that the levels of the three corresponding dNTPs were greatly elevated while the dCTP level decreased to the undetectable level (see “x”), compared to the untreated macrophages. This confirmed we established “single dNTP depletion” imbalance in macrophages.

Next, we investigated the impact of the single dCTP depletion imbalance established by the pretreatment of primary macrophages with the dA + dG + dT mixture on HIV-1 infection and reverse transcription. The single dCTP depletion decreased HIV-1 vector transduction (Fig. 2b), 2-LTR circle copy number (Fig. 2c), and production of HIV-1 89.6 (Fig. 2d), compared to the untreated macrophage control. These data demonstrate that, as seen in single dNTP elevation (Fig. 1), single dNTP depletion imbalance also inhibits HIV-1 infection in macrophages (Fig. 2).

### Effect of single dC treatment on HIV-1 replication

**Fig. 3:**
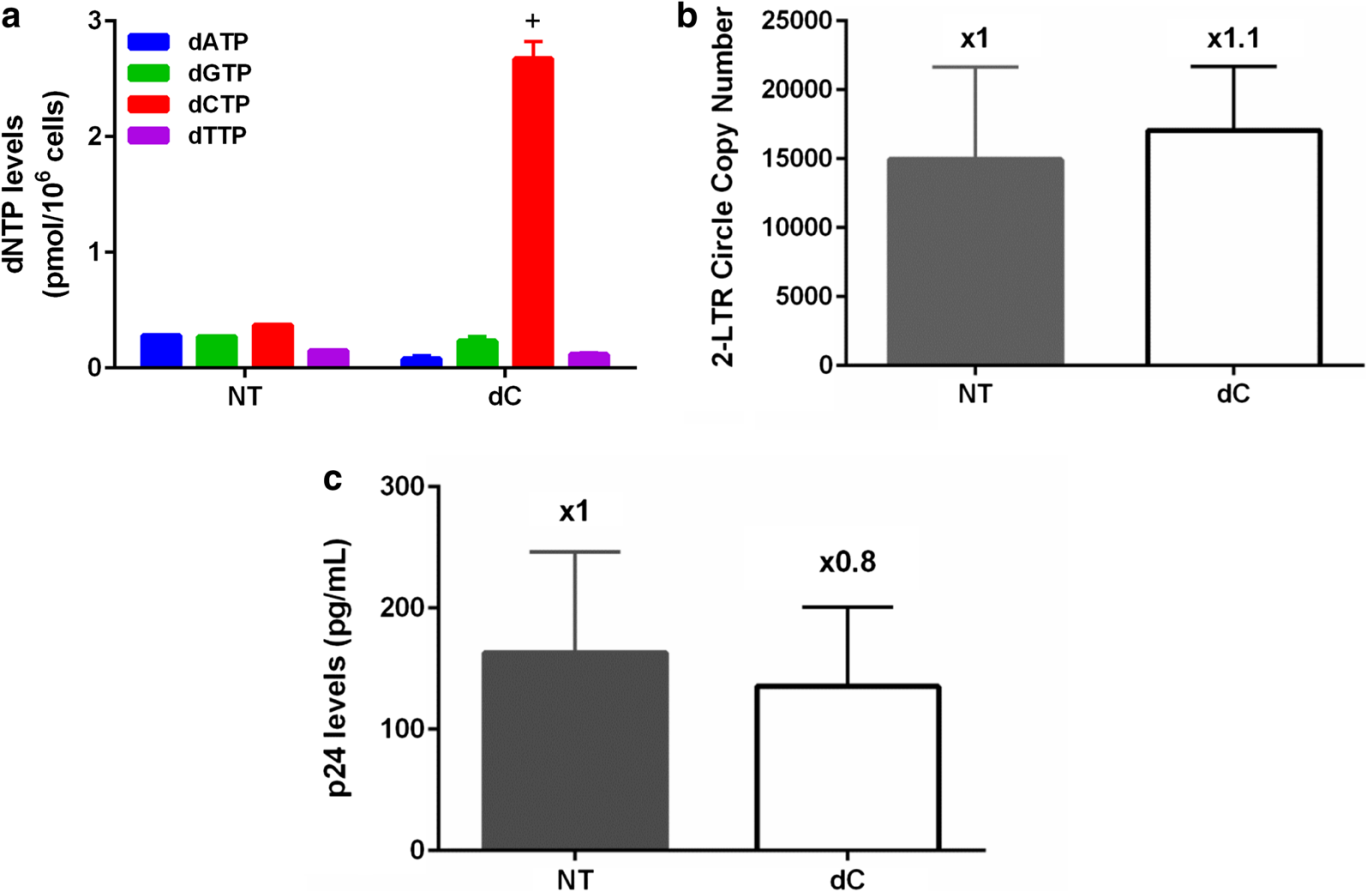
Effect of single dC treatment on HIV-1 replication in macrophage*s.* Human primary monocyte-derived macrophages prepared from four healthy donors were cultured with and without a mixture of dC (2.5 mM), and used for each experiment under the same condition described in Fig. 1: **a** dNTP level measurement, **b** 2-LTR circle DNA copy number assay, and **c** p24 level assay with infectious HIV-1 89.6 were conducted described in Fig. 1. “+” indicates the treated dCTP. *NT* no dN treatment. The data are the mean of three independent experiments and error bars represent the standard deviation from the mean

Interestingly, while the single dC treatment elevated dCTP concentration in macrophages (Fig. 3a), unlike the single dG, dA and dT treatments (Fig. 1a), the single dC treatment did not significantly affect the concentrations of other three dNTPs. Indeed, unlike other single dN bias treatments that reduced the HIV-1 infection, the dC treatment did not affect 2-LTR circle DNA copy number (Fig. 3b) and HIV-1 89.6 production (Fig. 3c), compared to the untreated macrophages (NT). Possibly, lack of reduction in other three untreated dNTP concentrations may contribute to the absence of the inhibitory effect of the single dC treatment against HIV-1 complete reverse transcription and viral production in macrophages.

### Biochemical simulation of HIV-1 RT mediated DNA synthesis under the induced dNTP pool imbalance

**Fig. 4:**
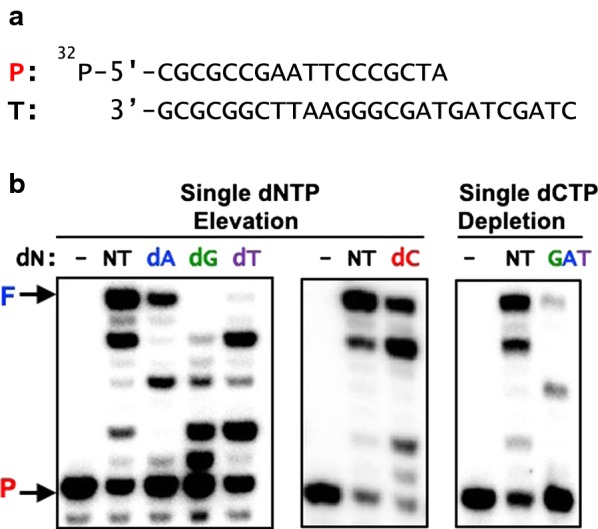
Biochemical simulations of HIV-1 RT mediated DNA synthesis under the induced dNTP pool imbalance. **a** 5′ ^32^P-labeled 18-mer DNA primer (P) annealed to a 26-mer template DNA (T) was used in the HIV-1 RT mediated primer extension reaction. The 5′ ^32^P-labeled primer (2 nM) annealed to the template was extended by purified HIV-1 RT protein for 5 min at 37 °C with the dNTP concentrations determined in macrophages with **b** single dNTP elevation (dG, dA, dT or dC treatment, from Figs. 1a and 3a) and single dNTP depletion (dG + dA + dT, “GAT” treatment from Fig. 2a). NT: dNTP concentrations from untreated macrophages (Fig. 1a). The concentrations of individual dNTPs in each reaction are shown in Additional file 1: Table S1. *F* fully extended 26-bp product, *P* 18-mer unextended primer

We biochemically simulated the HIV-1 RT mediated DNA synthesis under the single dNTP elevation and depletion conditions to mechanistically investigate the impact of these dNTP pool imbalances on HIV-1 reverse transcription. In this biochemical simulation, a 5′ ^32^P- labeled 18-mer DNA primer (P) annealed to a 26-mer DNA template (T) (Fig. 4a) was extended by purified HIV-1 RT protein at the dNTP concentrations observed in Figs. 1a and 3a (single dNTP elevation) as well as Fig. 2a (single dNTP depletion). See Additional file 1: Table S1 for each dNTP concentration used in these reactions.

First, we conducted a HIV-1 RT mediated primer extension reaction at the dNTP concentrations found in the untreated macrophages (“NT” in Fig. 4b), which generated > 80% primer extension in a 5 min incubation at 37 °C as calculated by the ratio of unextended primer (P) to fully extended 26-bp product (F). We then repeated the same RT-mediated primer extension reaction with the imbalanced dNTP concentrations calculated from the dNTP levels observed in single dNTP elevation (Figs. 1a and 3a) and depletion conditions (Fig. 2a). For the dNTPs that were reduced below the undetectable ranges of the RT based dNTP assay, we used 2–4 nM (detection limit). As shown in Fig. 4b (left panel), the 26-bp full length product (F) of the primer extension was significantly reduced at the three single dNTP elevation imbalance conditions induced by the single dG, dA or dT treatment, which also generated multiple RT pausing. Also, the larger amounts of the remaining unextened primer (P) can be seen in the reactions with the single dNTP elevation, confirming the inhibitory effect of the HIV-1 RT mediated DNA synthesis by these biased dNTP pools. Importantly, as discussed in Fig. 1a, while it was expected that biased dN treatments increased the corresponding dNTP concentrations (dGTP in dG treatment), we also observed that other untreated dNTP concentrations significantly decreased, compared to their levels in the untreated cells. The biochemical simulation data in Fig. 4b explains that the negative effect of the biased dN treatment against the untreated dNTPs (i.e. dATP, dCTP, and dTTP in the dG treatment) likely contributes to the reduction of the overall DNA synthesis.

As shown in Fig. 4b (right panel), we also observed the significant reduction of the full-length product in the HIV-1 RT mediated primer extension reactions at the dNTP concentration found in macrophages treated with the dG + dA + dT mixture (single dCTP depletion), which reduced HIV-1 production and reverse transcription (Fig. 2). However, in the biochemical simulation at the dNTP pools induced by the single dCTP elevation (Fig. 4b, middle panel), the full-length product amount was not significantly reduced, which explains the absence of the single dC treatment effect on HIV-1 production and reverse transcription in macrophages (Fig. 3). Overall, these biochemical simulations of HIV-1 reverse transcription under the various dNTP pool imbalance conditions give consistent mechanistic explanations about the effects of single dNTP elevation and depletion on HIV-1 reverse transcription and viral production in macrophages.

### Effect of induced dNTP imbalance on HIV-1 mutagenesis

**Fig. 5:**
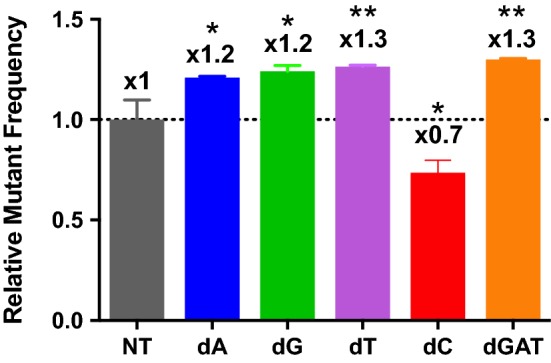
Effect of dNTP pool imbalances on HIV-1 mutant rate in macrophages. The HIV-1 mutant frequency was determined in human primary macrophages by using the pMIG HIV-1 vector based system as previously described [29, 30]. After transducing 4-h dN treated macrophages with pMIG HIV-1 vector, mutant frequency analysis was conducted by FACS. Mutant frequency was determined based on the percentage of mCherry and EGFP single positive cells, compared to the total number of infected cells. The equation used was (mCherry^+^EGFP^−^ cells + mCherry^−^EGFP^+^ cells) divided by total infected cells. The calculated mutant frequency in each treatment was normalized with the mutant frequency of the untreated macrophages (NT). The data are the mean of three independent experiments and error bars represent the standard deviation from the mean. *p-value < 0.05, **p-value < 0.01

We tested whether the dNTP pool imbalances established in macrophages by the treatments with the biased dNs affect HIV-1 mutagenesis. For this test, we employed a single round infection system of HIV-1 envelope-deficient vector pNL4-3 MIG which was previously engineered for measuring HIV-1 mutant frequency [29]. This vector co-expresses mCherry and EGFP proteins. Mutations in these two marker proteins can induce the loss of corresponding fluorescent proteins, therefore viral mutant frequency was determined based on the percentage of single positive cells compared to the total number of transduced cells: number of (mCherry^+^ EGFP^−^cells + mCherry^−^ EGFP^+^ cells) divided by the number of total transduced cells. In Fig. 5, compared to no treatment, single dNTP elevation and depletion imbalances elevated HIV-1 mutant frequency by 20–30% per infection cycle. Interestingly, the single dC treatment reduced the viral mutant frequency. This suggests that natural dCTP levels affect HIV-1 mutagenesis in macrophages, which was counteracted when the dCTP level was elevated.

### Effect of dN treatment on SIV_mac_239 infectivity in macrophages

**Fig. 6:**
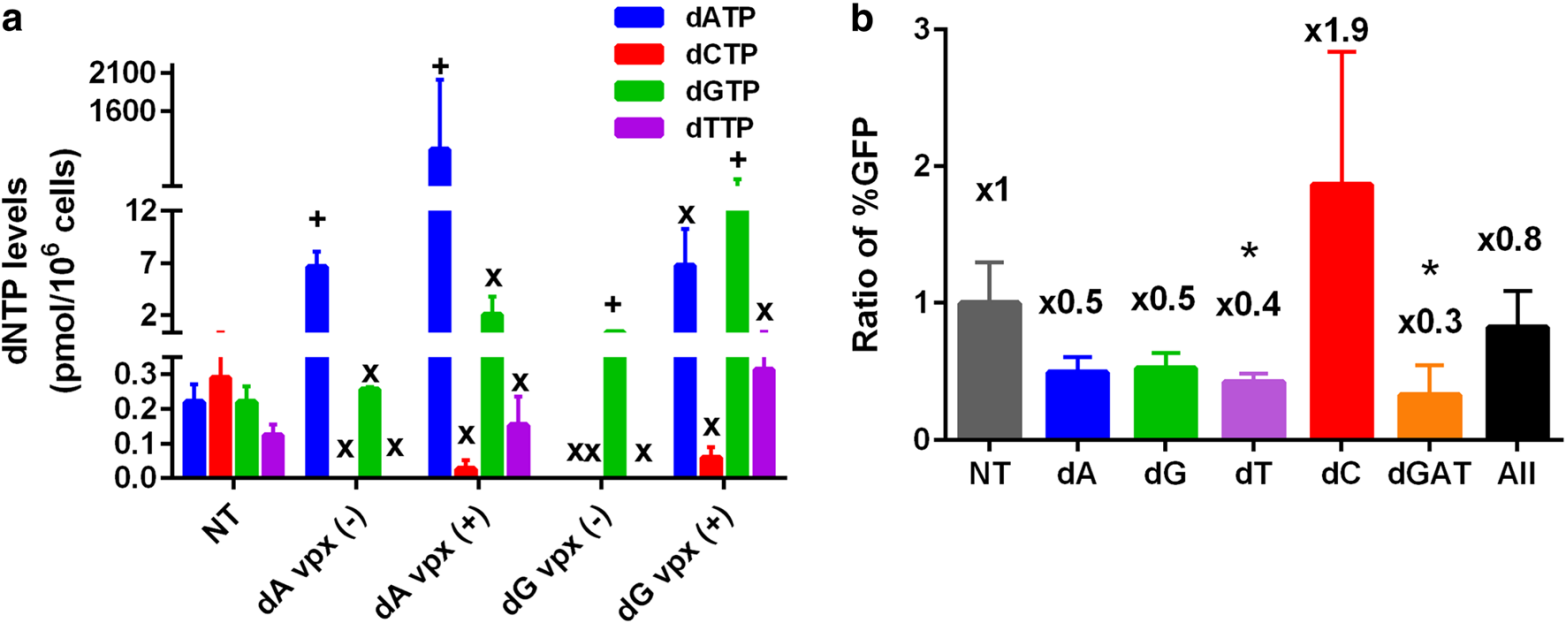
Effect of dNTP pool imbalances on transduction of SIV_mac_239 GFP vector in macrophages. Human primary monocyte-derived macrophages prepared from four healthy donors (**a**) dNTP levels were measured after macrophages were first treated with VLPs with (+) and without (−) Vpx for 12 h and then with 1 mM dA or dG for 4 h. The dNTP levels in these cells were determined by the RT-based dNTP assay. “+” indicates the treated dNTPs, and “x” indicates untreated dNTPs. *NT* No dN treatment. **b** Macrophages were treated with various biased dN as described in Fig. 1 (dG, dA or dT), 2 (dG + dA + dT), 3 (dC), or Additional file 1: Figure S1 “All” (dG, dA, dC or dT). The treated cells were transduced with SIV_mac_239-GFP vector, and transduction efficiency was measured via percent of GFP^+^ cells. The transduction efficiency for each treatment was normalized to the transduction efficiency with no dN treatment. The data are the mean of three independent experiments and error bars represent the standard deviation from the mean. *p-value < 0.05

Unlike HIV-1, many SIV and HIV-2 strains are capable of counteracting SAMHD1 mediated dNTP depletion in nondividing macrophages. SIV_mac_239 Vpx proteosomally degrades host SAMHD1 dNTPase, elevating cellular dNTP levels and accelerating the reverse transcription kinetics of these lentiviruses in macrophages. Since Vpx can elevate all four dNTPs in macrophages [15], it is highly likely that the concentration discrepancy among four dNTPs induced by the biased dN treatment would be much smaller in macrophages pre-exposed to Vpx than macrophages treated only with the biased dNs. In addition, because Vpx elevates all four dNTPs, Vpx likely avoids the reduction of the untreated dNTP levels that blocks the overall DNA synthesis and viral reverse transcription. First, we measured the dNTP levels in macrophages treated with dA or dG treatment and with virus like particles (VLPs) with (+) or without (−) Vpx. As shown in Fig. 6a, all four dNTPs including dCTP were able to be detected even with these biased dN treatments in the macrophages pretreated with Vpx [dA-Vpx (+) or dG-Vpx (+)], whereas most of the untreated dNTPs were not detected (see “x”) in the macrophages treated with biased dNs without Vpx [dA-Vpx (−) and dG-Vpx (−)]. Next, we tested the effect of the biased dN treatments on the transduction of SIV_mac_239 based GFP vector encoding Vpx in macrophages. We pretreated human primary macrophages with single bias dN, and transduced these macrophages with SIV_mac_239 GFP vector. As shown in Fig. 6b, the biased dN treatments display smaller reductions of the SIV GFP vector transduction, compared to HIV-1 GFP vector (see Figs. 1 and 2). Overall, the data shown in Fig. 6 support that the Vpx-mediated SAMHD1 degradation and elevation of all four dNTP levels minimizes the effect of the dNTP pool bias induced inhibitory effect against lentiviral replication in macrophages.

## Discussion

dNTP levels in cells are never equal, and the dNTP pool imbalance contributes to mutation synthesis during genomic DNA replication [7, 8]. dNTP incorporation kinetics during enzymatic DNA polymerization is affected by various elements such as dNTP substrate availability, template sequences, and DNA polymerases [3–6]. In dividing cells with the high dNTP concentrations, where DNA synthesis rate is close to *V*_max_, the dNTP bias likely minimally affects the kinetic variation of each dNTP incorporation and mutation synthesis [15, 22]. HIV-1 always replicates under very limited dNTP pool conditions (20–40 nM) and below the *K*_m_ value of HIV-1 RT in macrophages due to the host SAMHD1 dNTPase [15]. In order to investigate the effect of the dNTP imbalance on HIV-1 replication effectively, we employed macrophages with limited dNTP pools. Any variation of dNTP substrate availability and pool imbalance at these extremely low dNTP concentrations, below the *K*_m_ value of HIV-1 RT, directly affects the incorporation of each dNTP.

As we expected, the treatments of macrophages with biased dNs generated large dNTP pool imbalances by the elevation of the corresponding dNTPs to the treated dNs. However, even greater dNTP imbalances by the biased dN treatments were established by unexpected decreases of the non-treated dNTPs. Indeed, in the case of the single dGTP elevation, the other three non-treated dNTPs were decreased below the detection limit of our dNTP assay (Fig. 1), and in the case of the dA + dG + dT treatment (Fig. 2), no dCTP was detected in the assay. We suspect that the unexpected decreases of the non-treated dNTPs may result from the monopolization of cellular enzymes involved in triphosphate form synthesis (i.e. deoxynucleoside/deoxynucleotide kinases) by the treated dNs. The excess amount of the treated dNs could significantly limit the cellular synthesis of other non-treated dNTPs.

In our infectivity experiments, we observed two different impacts made by the biased dN treatments in macrophages. First, the dNTP pool imbalances inhibited HIV-1 infection and reverse transcription. One reason for this inhibitory effect is likely because the unexpected decreases of the non-treated dNTPs. As discussed above, any changes in the dNTP substrate concentrations below the *K*_m_ value of HIV-1 RT directly affect the DNA synthesis rates. Also, the decreases of the non-treated dNTP concentrations should interfere with the overall HIV-1 reverse transcription kinetics in macrophages. Second, as we expected, the dNTP pool imbalances induced the elevation of HIV-1 mutations by 20–30% per a single infection cycle. In the HIV-1 vector-based assay system, we can detect the mutations generated only in the individual viruses that successfully completed reverse transcription even though the biased dN treatments significantly blocked the HIV-1 vector transduction. In other words, in addition to the reduction of the non-treated dNTPs, the forced mutation synthesis by the dNTP pool imbalance can also induce the failure of the completion of reverse transcription. Mutation synthesis processes can significantly slow down processive DNA synthesis. More specifically, a single mutation synthesis during enzymatic DNA polymerization consists of two sequential sub-steps: (1) misincorporation (incorporation of wrong dNTPs) that generates mismatches and (2) mismatch extension [31]. Both misincorporation and mismatch extension are kinetically much slower, compared to correct dNTP incorporation and matched primer extension, respectively [31]. Even though the highly elevated treated dNTP concentrations (i.e. dGTP in the dG treatment) can force the misincorporation step, the second mismatch extension step must become drastically hindered if the dNTPs necessary for the next mismatch extension are the unexpectedly further decreased non-treated dNTPs. Basically, it is possible that the viruses, which undergo multiple mutation synthesis events, may fail to complete reverse transcription. This would not be counted in our mutant rate assay because our assay counts only the viruses that complete their reverse transcription step. This argues that the combination of the unexpected decrease of the untreated dNTP concentrations and elevated mutation synthesis could explain the reduction of the overall HIV-1 infectivity and production in macrophages treated with the biased dNs.

Importantly, we previously reported that HIV-1 RT is uniquely efficient in mismatch extension, compared to high fidelity murine leukemia virus (MuLV) RT. This efficient mismatch extension capability of HIV-1 RT allows HIV-1 to complete reverse transcription even after mutation synthesis that interferes with the processive DNA synthesis, generating live and mutant viruses [32]. However, the reverse transcription process of MuLV is likely terminated after misincorporation by MuLV RT because MuLV RT is almost incapable of extending a mismatch primer, failing to produce live and mutant viruses. In our current study, even with the highly efficient mismatch extension capability of HIV-1 RT, HIV-1 infection was significantly inhibited by large dNTP pool imbalance conditions, possibly because these conditions can induce multiple mutation synthesis events per infection.

dNTP pool imbalance could not be effectively established in dividing/activated CD4^+^ T cells because the treatment of this cell type with biased dNs at the applied concentrations induces cell death. Furthermore, activated CD4^+^ T cells already have saturating dNTP levels for HIV-1 RT (much higher than *V*_max_ of HIV-1 RT), which likely generates minimal effect of the dNTP pool imbalance on HIV-1 replication. This possibility is supported by our observation of the minimal effect of the dNTP pool imbalance on SIV_mac_239 vector that replicates at high dNTP concentrations even in macrophages due to Vpx that degrades host SAMHD1 dNTPase. Interestingly, in Fig. 6, upon the addition of dA or dG with SAMHD1 present (Vpx −), we see that SAMHD1 keeps the dA or dG level lower than when SAMHD1 is not present (Vpx +). We propose that the dA and dG levels are increasing in the SAMHD1 present (Vpx −) conditions because SAMHD1 could be saturated by the excess dNTP.

## Conclusion

Overall, based on our investigations of the effect of the biased dN treatments on the transduction efficiency and 2-LTR circle copy number of HIV-1 vector, production of infectious HIV-1 89.6, viral mutant rate, and transduction of SIV_mac_239-based vector as well as biochemical DNA synthesis simulations, we conclude that the dNTP pool imbalance can induce both replication kinetic block and mutation synthesis of HIV-1 in macrophages.

## Supplementary information

**Additional file 1: Figure S1.** Effect of treatment with all 4 dNs on dNTP levels and HIV-1 infection in macrophages. **Table S1.** Concentration of dNTPs used in biochemical simulations (Fig. 4). **Table S2.** Raw %GFP numbers (Figs. 1b, 2b, and 6b).

## Data Availability

All data generated or analyzed during this study are included in this published article and its Additional file.

## Abbreviations

HIV-1: human immunodeficiency virus type 1
HIV-2: human immunodeficiency type 2
SIV: simian immunodeficiency virus
Vpx: viral protein X
SAMHD1: SAM domain and HD domain containing protein 1
RT: reverse transcriptase
dNTP: deoxynucleoside triphosphate
dNs: deoxynucleosides
VLP: virus like particles
