# Supplementary material for: Effect of induced dNTP pool imbalance on HIV-1 reverse transcription in macrophages
Source: Retrovirology. 2019 Oct 26;16:29. doi: 10.1186/s12977-019-0491-0 (PMC6815395; doi:10.1186/s12977-019-0491-0)
Supplement: Supplementary file 1 — Additional file 1: Figure S1. Effect of treatment with all 4 dNs on dNTP levels and HIV-1 infection in macrophages. Table S1. Concentration of dNTPs used in biochemical simulations (Fig. 4). Table S2. Raw %GFP numbers (Figs. 1b, 2b, and 6b). [file 12977_2019_491_MOESM1_ESM.docx]

**Figure S1**

| **(A)** | **(B)** |
| --- | --- |
|  |  |
|  | **(C)** |
|  |  |

**Figure S1:** **Effect of treatment with all 4 dNs on dNTP levels and HIV-1 infection in macrophages*.*** All experiments were conducted in human primary monocyte-derived macrophages prepared from four healthy donors. dA, dC, dG and dT treatments were at 1 mM. **(A)** dNTP levels were measured after macrophages were treated with all 4 dN treatments. After 12 hours, the dNTP levels in these cells were determined by the RT-based dNTP assay. **(B)** HIV-1 vector transduction efficiency was measured after macrophages were pretreated with dNs for 4 h, and then transduced with an equal amount of HIV-GFP vector. The transduced cells were collected after 6 days, and the percent of the GFP^+^ cells was determined by FACS. **(C)** p24 antigen levels were measured by ELISA. Macrophages were treated with dN treatment for 4 h followed by dual tropic HIV-1 89.6 infection. Supernatant was collected 6 days after infection. Supernatant was used for a HIV-1 p24 ELISA. The data are the mean of two independent experiments and error bars represent the standard deviation from the mean. **p-value* <0.05, ****p-value* <0.001.

**Table S1: Concentration of dNTPs used in biochemical simulations (Figure 4).**

| Treatments | Concentration (nM) | | | |
| --- | --- | --- | --- | --- |
|  | dATP | dGTP | dCTP | dTTP |
| NT | 85 | 85 | 112 | 47 |
| dA | 1494 | 102 | 4 | 33 |
| dG | 4 | 1046 | 4 | 4 |
| dT | 4 | 96 | 12 | 217 |
| dC | 31 | 90 | 1029 | 46 |
| dGAT | 3673 | 2 | 1328 | 1074 |

**Table S2: Raw %GFP numbers (Figure 1B, 2B, and 6B).**

| Figure 1B | | | |  |  |  |
| --- | --- | --- | --- | --- | --- | --- |
| NT | dA | dG | dT |  |  |  |
| 3.26 | 0.2 | 1.14 | 0.18 |  |  |  |
| 6.97 | 1.15 | 0.44 | 0.81 |  |  |  |
| 6.08 | 1.48 | 1.18 | 0.71 |  |  |  |
|  |  |  |  |  |  |  |
| Figure 2B | |  |  |  |  |  |
| NT | dGAT |  |  |  |  |  |
| 3.26 | 1.35 |  |  |  |  |  |
| 6.97 | 1.08 |  |  |  |  |  |
| 6.08 | 0.76 |  |  |  |  |  |
|  |  |  |  |  |  |  |
| Figure 6B | | | | | | |
| NT | dA | dG | dT | dC | dGAT | All |
| 21.6 | 9.88 | 8.7 | 7.81 | 58.53 | 7.19 | 22.57 |
| 15.54 | 9 | 12.6 | 9.96 | 46.73 | 12 | 19.88 |
| 28.49 | 13.59 | 13.22 | 10.28 | 17.28 | 2.6 | 11.54 |
